# Methyl (3*S*,10b’*S*)-5-chloro-9′-fluoro-1-methyl-2-oxo-5′-phenyl-10b’*H*-spiro­[indoline-3,1′-pyrazolo­[3,2-*a*]iso­quinoline]-2′-carboxyl­ate

**DOI:** 10.1107/S1600536813011549

**Published:** 2013-05-04

**Authors:** Piskala Subburaman Kannan, PanneerSelvam Yuvaraj, Boreddy Siva Rami Reddy, Rajamani Raja, Arunachalathevar SubbiahPandi

**Affiliations:** a Department of Physics, S.M.K. Fomra Institute of Technology, Thaiyur, Chennai 603 103, India; b Industrial Chemistry Laboratory, Central Leather Research Institute, Adyar, Chennai 600 020, India; c Department of Physics, Presidency College (Autonomous), Chennai 600 005, India

## Abstract

In the title compound, C_27_H_19_ClFN_3_O_3_, the pyrazole ring has a twist conformation and the six-membered ring to which it is fused has a screw-boat conformation. The mean plane of the pyrazole ring is inclined to the 2-methyl­indoline ring by 85.03 (9) and by 28.17 (8)° to the mean plane of the iso­quinoline ring system. In the crystal, mol­ecules are linked by pairs of C—H⋯F hydrogen bonds, forming inversion dimers. These dimers are linked *via* C—H⋯O hydrogen bonds, forming a two-dimensional network lying parallel to (10-1).

## Related literature

For the biological activity of pyrazoles, see: Huang *et al.* (1996 ▶); Li *et al.* (2005 ▶); Patel *et al.* (1990 ▶); Zhao *et al.* (2001 ▶). For the crystal structures of pyrazoles, see: Manivel *et al.* (2009 ▶); Khan *et al.* (2010*a* ▶,*b* ▶,*c* ▶). For the crystal structure of an isoquinazole, see: Hathwar *et al.* (2008 ▶). For the biological activity of fused iso­quinoline compounds, see: Aubry *et al.* (2004 ▶); Marco *et al.* (2005 ▶); Reddy *et al.* (1999 ▶). For related structures, see: Chen & Wu (2010 ▶); Ye *et al.* (2010 ▶); Yu *et al.* (2011*a* ▶,*b* ▶). For ring conformations, see: Cremer & Pople (1975 ▶).
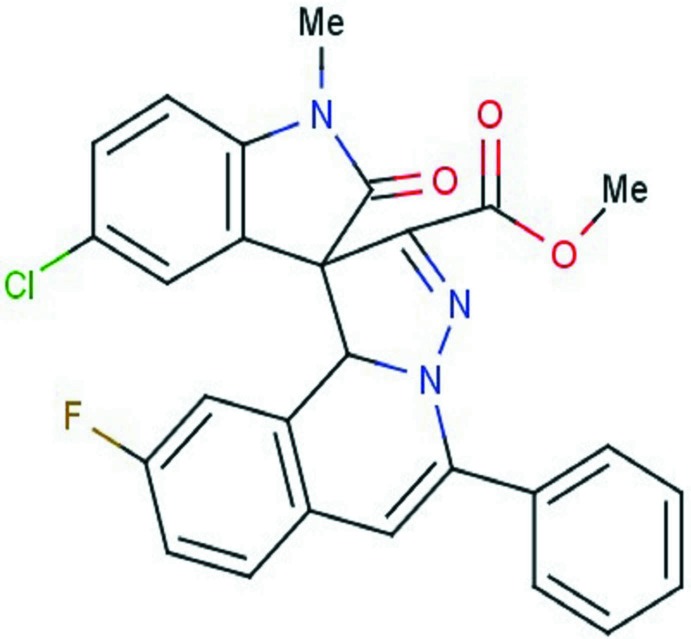


## Experimental

### 

#### Crystal data

C_27_H_19_ClFN_3_O_3_
*M* *_r_* = 487.90
Monoclinic, 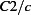

*a* = 15.1203 (3) Å
*b* = 21.1088 (5) Å
*c* = 15.6334 (3) Å
β = 112.977 (1)°
*V* = 4593.85 (17) Å^3^
*Z* = 8
Mo *K*α radiation
μ = 0.21 mm^−1^
*T* = 293 K
0.30 × 0.25 × 0.20 mm

#### Data collection

Bruker SMART APEXII area-detector diffractometer
Absorption correction: multi-scan (*SADABS*; Bruker, 2008 ▶) *T*_min_ = 0.940, *T*_max_ = 0.959
22581 measured reflections
5703 independent reflections
4241 reflections with *I* > 2σ(*I*)
*R*_int_ = 0.028

#### Refinement

*R*[*F* ^2^ > 2σ(*F* ^2^)] = 0.041
*wR*(*F* ^2^) = 0.123
*S* = 1.03
5703 reflections
318 parameters
H-atom parameters constrained
Δρ_max_ = 0.25 e Å^−3^
Δρ_min_ = −0.30 e Å^−3^

### 

Data collection: *APEX2* (Bruker, 2008 ▶); cell refinement: *SAINT* (Bruker, 2008 ▶); data reduction: *SAINT*; program(s) used to solve structure: *SHELXS97* (Sheldrick, 2008 ▶); program(s) used to refine structure: *SHELXL97* (Sheldrick, 2008 ▶); molecular graphics: *ORTEP-3 for Windows* (Farrugia, 2012 ▶); software used to prepare material for publication: *SHELXL97* and *PLATON* (Spek, 2009 ▶).

## Supplementary Material

Crystal structure: contains datablock(s) global, I. DOI: 10.1107/S1600536813011549/su2581sup1.cif

Structure factors: contains datablock(s) I. DOI: 10.1107/S1600536813011549/su2581Isup2.hkl

Supplementary material file. DOI: 10.1107/S1600536813011549/su2581Isup3.cml

Additional supplementary materials:  crystallographic information; 3D view; checkCIF report

## Figures and Tables

**Table 1:** Hydrogen-bond geometry (Å, °)

*D*—H⋯*A*	*D*—H	H⋯*A*	*D*⋯*A*	*D*—H⋯*A*
C27—H27*B*⋯F1^i^	0.96	2.52	3.226 (3)	130
C14—H14⋯O1^ii^	0.93	2.50	3.402 (2)	163

Symmetry codes: (i) 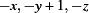; (ii) 
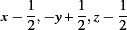
.

**Fig. 1.**
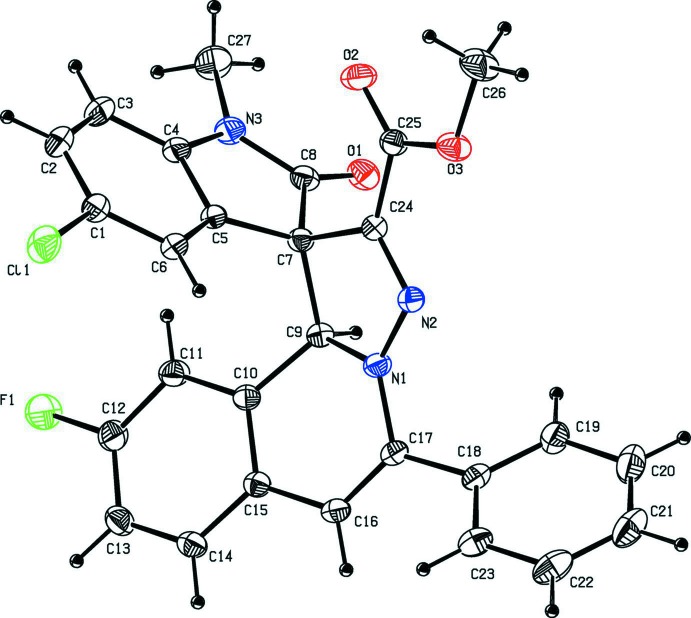
The molecular structure of the title molecule, with atom labelling. Displacement ellipsoids are drawn at the 30% probability level.

**Fig. 2.**
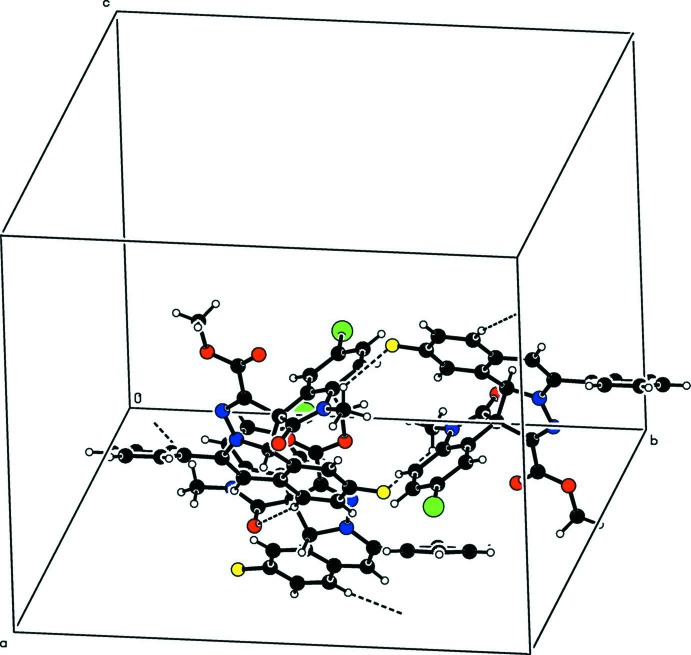
The partial view of the crystal packing of the title compound. The C-H···F and C-H···O hydrogen bonds are shown as dashed lines (see Table 1 for details; Cl green, F yellow).

## supplementary crystallographic information

### Comment

Pyrazole and its derivatives are a class of important five-membered heterocycle compounds with two adjacent nitrogen atoms. During the past years considerable evidence has been accumulated to demonstrate the biological efficacy of pyrazole derivatives, including antibacterial (Patel *et al.*, 1990), antifungal (Zhao *et al.*, 2001), herbicidal (Li *et al.*, 2005), insecticidal (Huang *et al.*, 1996) and other biological activities. A number of pyrazole-containing compounds have been successfully commercialized, such as the blockbuster drugs Viagra, Celebrex, and Acomplia.

Among the family of isoquinolines, the fused isoquinolines have attracted much attention owing to their biological activities including potent inhibitor of human topoisomerase I and selective inhibition against HIV-1 integrase in vitro (Aubry *et al.*, 2004; Marco *et al.*, 2005; Reddy *et al.*, 1999). In view of the diverse applications of this class of compounds, and continuing our research on the synthesis and crystal structure analysis of similar compounds (Manivel *et al.*, 2009; Khan *et al.*, 2010*a*,*b*,*c*; Hathwar *et al.*, 2008), we report herein on the crystal structure of the new title isoquinoline pyrazole compound.

The molecular structure and atom connectivity of the title compound are illustrated in Fig. 1. The isoquinoline ring system (C9-C17/N1), the methyldihydroindole ring system (N3/C1-C8) and the pyrazole ring (N1-N2/C7/C9/C24) are relatively planar, with maximum deviations from their mean planes of -0.212 Å for atom N1, -0.041 Å for C5 and 0.111 Å for C9, respectively.

The pyrazole ring mean plane forms a dihedral angle of 85.03 (9) ° with the methyldihydroindole ring system. This clearly shows that the pyrazole ring is almost perpendicular to the methyldihydroindole ring system. The dihedral angle between mean planes of the pyrazole ring and the isoquinoline ring system is 28.17 (8)°.

The pyrazole ring is twisted on bond C7-C9 with puckering parameters of q_2_ = 0.1879 (2) Å, φ = 129.28 (5)° [Cremer & Pople, 1975.

In the crystal, molecules are linked by a pair of C—H···F hydrogen bonds forming inversion dimers (Table 1). These dimers are linked *via* C—H···O hydrogen bonds forming a two-dimensional network lying parallel to the (101) plane (Table 1 and Fig. 2).

### Experimental

General experimental procedure for the silver triflate-catalyzed tandem reaction of N'-(2-alkynylbenzylidene) hydrazide with methyleneindolinones: A mixture of N'-(2-alkynylbenzylidene) hydrazide (0.3 mmol) and AgOTf (10 mol%) in DCE (2.0 mL) was heated at 333 K with vigorous stirring for 1 hour. Then, the methyleneindolinone (0.45 mmol, 1.5 equiv), Cs_2_CO_3_ (0.9 mmol, 3.0 equiv) and toluene (2.0 mL) were added. The reaction mixture was refluxed at 353 K until completion of the reaction. The reaction mixture was diluted with ethyl acetate (5.0 mL) and quenched with water (5.0 mL). The organic layer was washed with brine, dried over Na_2_SO_4_ and concentrated under reduced pressure. The residue was purified by column chromatography using ethyl acetate and hexane (3:7) as an elutent on neutral alumina to provide the desired product. Block-like crystals, suitable for X-ray diffraction analysis, were obtained by slow evaporation of a solution in ethyl acetate at room temperature.

### Refinement

All H atoms were fixed geometrically and allowed to ride on their parent C atom: C—H 0.93–0.97 Å with *U*_iso_(H) = 1.5*U*_eq_(C-methyl) and = 1.2*U*_eq_(C) for other H atoms. The positions of the methyl hydrogens were optimized rotationally.

### Crystal data

**Table:**

C_27_H_19_ClFN_3_O_3_	*F*(000) = 2016
*M**_r_* = 487.90	*D*_x_ = 1.411 Mg m^−^^3^
Monoclinic, *C*2/*c*	Mo *K*α radiation, λ = 0.71073 Å
Hall symbol: -C 2yc	Cell parameters from 5703 reflections
*a* = 15.1203 (3) Å	θ = 1.8–28.3°
*b* = 21.1088 (5) Å	µ = 0.21 mm^−^^1^
*c* = 15.6334 (3) Å	*T* = 293 K
β = 112.977 (1)°	Block, colourless
*V* = 4593.85 (17) Å^3^	0.30 × 0.25 × 0.20 mm
*Z* = 8	

### Data collection

**Table:**

Bruker SMART APEXII area-detector diffractometer	5703 independent reflections
Radiation source: fine-focus sealed tube	4241 reflections with *I* > 2σ(*I*)
Graphite monochromator	*R*_int_ = 0.028
ω and φ scans	θ_max_ = 28.3°, θ_min_ = 1.8°
Absorption correction: multi-scan (*SADABS*; Bruker, 2008)	*h* = −19→20
*T*_min_ = 0.940, *T*_max_ = 0.959	*k* = −26→28
22581 measured reflections	*l* = −20→20

### Refinement

**Table:**

Refinement on *F*^2^	Primary atom site location: structure-invariant direct methods
Least-squares matrix: full	Secondary atom site location: difference Fourier map
*R*[*F*^2^ > 2σ(*F*^2^)] = 0.041	Hydrogen site location: inferred from neighbouring sites
*wR*(*F*^2^) = 0.123	H-atom parameters constrained
*S* = 1.03	*w* = 1/[σ^2^(*F*_o_^2^) + (0.0562*P*)^2^ + 2.5788*P*] where *P* = (*F*_o_^2^ + 2*F*_c_^2^)/3
5703 reflections	(Δ/σ)_max_ < 0.001
318 parameters	Δρ_max_ = 0.25 e Å^−^^3^
0 restraints	Δρ_min_ = −0.30 e Å^−^^3^

### Special details

**Table:**

Geometry. All esds (except the esd in the dihedral angle between two l.s. planes) are estimated using the full covariance matrix. The cell esds are taken into account individually in the estimation of esds in distances, angles and torsion angles; correlations between esds in cell parameters are only used when they are defined by crystal symmetry. An approximate (isotropic) treatment of cell esds is used for estimating esds involving l.s. planes.
Refinement. Refinement of F^2^ against ALL reflections. The weighted R-factor wR and goodness of fit S are based on F^2^, conventional R-factors R are based on F, with F set to zero for negative F^2^. The threshold expression of F^2^ > 2sigma(F^2^) is used only for calculating R-factors(gt) etc. and is not relevant to the choice of reflections for refinement. R-factors based on F^2^ are statistically about twice as large as those based on F, and R- factors based on ALL data will be even larger.

### Fractional atomic coordinates and isotropic or equivalent isotropic displacement parameters (Å^2^)

**Table:**

	*x*	*y*	*z*	*U*_iso_*/*U*_eq_	
C1	−0.17222 (12)	0.36693 (8)	0.05994 (11)	0.0504 (4)	
C2	−0.13286 (15)	0.42416 (9)	0.09683 (14)	0.0633 (5)	
H2	−0.1682	0.4526	0.1162	0.076*	
C3	−0.04086 (15)	0.44005 (9)	0.10548 (14)	0.0640 (5)	
H3	−0.0137	0.4788	0.1306	0.077*	
C4	0.00932 (12)	0.39673 (7)	0.07581 (11)	0.0472 (4)	
C5	−0.03056 (11)	0.33864 (7)	0.03903 (9)	0.0391 (3)	
C6	−0.12155 (11)	0.32268 (7)	0.03097 (10)	0.0428 (3)	
H6	−0.1483	0.2837	0.0070	0.051*	
C7	0.04406 (10)	0.30004 (7)	0.02057 (9)	0.0374 (3)	
C8	0.13007 (11)	0.34625 (7)	0.04972 (10)	0.0432 (3)	
C9	0.02260 (11)	0.27304 (7)	−0.07765 (10)	0.0380 (3)	
H9	0.0821	0.2753	−0.0885	0.046*	
C10	−0.05599 (11)	0.30133 (7)	−0.16069 (10)	0.0390 (3)	
C11	−0.06588 (13)	0.36633 (8)	−0.17251 (11)	0.0499 (4)	
H11	−0.0280	0.3938	−0.1261	0.060*	
C12	−0.13297 (14)	0.38943 (8)	−0.25436 (12)	0.0560 (4)	
C13	−0.19128 (13)	0.35141 (9)	−0.32462 (12)	0.0539 (4)	
H13	−0.2365	0.3687	−0.3787	0.065*	
C14	−0.18089 (11)	0.28690 (8)	−0.31272 (11)	0.0465 (4)	
H14	−0.2198	0.2603	−0.3598	0.056*	
C15	−0.11317 (10)	0.26033 (7)	−0.23147 (10)	0.0389 (3)	
C16	−0.09822 (11)	0.19251 (7)	−0.22125 (10)	0.0419 (3)	
H16	−0.1288	0.1667	−0.2726	0.050*	
C17	−0.04200 (11)	0.16548 (7)	−0.14060 (10)	0.0396 (3)	
C18	−0.02320 (12)	0.09669 (7)	−0.12735 (11)	0.0444 (3)	
C19	0.06896 (14)	0.07361 (8)	−0.08048 (13)	0.0564 (4)	
H19	0.1201	0.1015	−0.0544	0.068*	
C20	0.08479 (18)	0.00873 (10)	−0.07247 (16)	0.0731 (6)	
H20	0.1468	−0.0066	−0.0413	0.088*	
C21	0.0103 (2)	−0.03290 (10)	−0.10988 (17)	0.0782 (7)	
H21	0.0215	−0.0763	−0.1035	0.094*	
C22	−0.0807 (2)	−0.01051 (10)	−0.15659 (17)	0.0778 (6)	
H22	−0.1313	−0.0389	−0.1824	0.093*	
C23	−0.09844 (15)	0.05428 (9)	−0.16584 (13)	0.0605 (5)	
H23	−0.1606	0.0691	−0.1978	0.073*	
C24	0.06465 (10)	0.23823 (7)	0.07479 (10)	0.0381 (3)	
C25	0.10235 (11)	0.23704 (8)	0.17662 (10)	0.0438 (3)	
C26	0.14650 (18)	0.17390 (11)	0.31036 (13)	0.0757 (6)	
H26A	0.2121	0.1879	0.3336	0.114*	
H26B	0.1442	0.1306	0.3281	0.114*	
H26C	0.1107	0.1998	0.3359	0.114*	
C27	0.16737 (16)	0.45421 (10)	0.11482 (18)	0.0786 (6)	
H27A	0.2259	0.4468	0.1060	0.118*	
H27B	0.1814	0.4588	0.1799	0.118*	
H27C	0.1375	0.4922	0.0828	0.118*	
N1	0.00398 (10)	0.20592 (6)	−0.06590 (8)	0.0428 (3)	
N2	0.03649 (9)	0.18827 (6)	0.02346 (8)	0.0406 (3)	
N3	0.10284 (10)	0.40106 (6)	0.07812 (10)	0.0519 (3)	
O1	0.20707 (8)	0.33595 (6)	0.04558 (8)	0.0542 (3)	
O2	0.12795 (11)	0.28419 (6)	0.22236 (8)	0.0679 (4)	
O3	0.10528 (9)	0.17891 (6)	0.21056 (8)	0.0561 (3)	
Cl1	−0.28762 (4)	0.34823 (3)	0.05025 (4)	0.07239 (17)	
F1	−0.13982 (11)	0.45332 (5)	−0.26685 (8)	0.0889 (4)	

### Atomic displacement parameters (Å^2^)

**Table:**

	*U*^11^	*U*^22^	*U*^33^	*U*^12^	*U*^13^	*U*^23^
C1	0.0494 (9)	0.0546 (9)	0.0479 (8)	0.0061 (7)	0.0196 (7)	−0.0052 (7)
C2	0.0652 (12)	0.0565 (11)	0.0661 (11)	0.0122 (9)	0.0234 (9)	−0.0172 (9)
C3	0.0694 (13)	0.0430 (9)	0.0703 (12)	0.0002 (8)	0.0171 (10)	−0.0204 (8)
C4	0.0492 (9)	0.0393 (8)	0.0441 (8)	−0.0023 (7)	0.0085 (7)	−0.0063 (6)
C5	0.0443 (8)	0.0356 (7)	0.0335 (7)	0.0010 (6)	0.0111 (6)	−0.0034 (5)
C6	0.0457 (8)	0.0400 (7)	0.0421 (8)	0.0000 (6)	0.0164 (6)	−0.0041 (6)
C7	0.0373 (7)	0.0363 (7)	0.0356 (7)	−0.0032 (6)	0.0108 (6)	−0.0029 (5)
C8	0.0424 (8)	0.0425 (8)	0.0378 (7)	−0.0061 (6)	0.0080 (6)	0.0005 (6)
C9	0.0397 (7)	0.0380 (7)	0.0359 (7)	−0.0041 (6)	0.0144 (6)	−0.0034 (5)
C10	0.0412 (8)	0.0420 (7)	0.0356 (7)	−0.0016 (6)	0.0170 (6)	−0.0004 (6)
C11	0.0630 (10)	0.0433 (8)	0.0394 (8)	−0.0029 (7)	0.0157 (7)	−0.0006 (6)
C12	0.0731 (12)	0.0450 (9)	0.0482 (9)	0.0071 (8)	0.0220 (9)	0.0073 (7)
C13	0.0524 (10)	0.0635 (11)	0.0414 (8)	0.0107 (8)	0.0134 (7)	0.0082 (7)
C14	0.0382 (8)	0.0600 (10)	0.0391 (7)	−0.0022 (7)	0.0127 (6)	−0.0030 (7)
C15	0.0366 (7)	0.0461 (8)	0.0363 (7)	−0.0014 (6)	0.0167 (6)	−0.0021 (6)
C16	0.0413 (8)	0.0462 (8)	0.0387 (7)	−0.0068 (6)	0.0160 (6)	−0.0104 (6)
C17	0.0424 (8)	0.0391 (7)	0.0399 (7)	−0.0038 (6)	0.0189 (6)	−0.0083 (6)
C18	0.0566 (9)	0.0383 (7)	0.0425 (8)	−0.0018 (7)	0.0238 (7)	−0.0074 (6)
C19	0.0611 (11)	0.0460 (9)	0.0634 (11)	0.0044 (8)	0.0256 (9)	−0.0029 (8)
C20	0.0887 (15)	0.0547 (11)	0.0832 (14)	0.0205 (11)	0.0415 (13)	0.0057 (10)
C21	0.118 (2)	0.0417 (10)	0.0919 (16)	0.0034 (12)	0.0594 (15)	−0.0057 (10)
C22	0.1026 (18)	0.0479 (11)	0.0902 (16)	−0.0224 (11)	0.0456 (14)	−0.0230 (10)
C23	0.0689 (12)	0.0498 (10)	0.0619 (11)	−0.0118 (9)	0.0247 (9)	−0.0152 (8)
C24	0.0382 (7)	0.0378 (7)	0.0375 (7)	0.0009 (6)	0.0141 (6)	−0.0009 (5)
C25	0.0419 (8)	0.0494 (9)	0.0379 (7)	0.0009 (7)	0.0133 (6)	−0.0012 (6)
C26	0.0905 (16)	0.0884 (15)	0.0405 (9)	0.0013 (12)	0.0171 (10)	0.0156 (9)
C27	0.0661 (13)	0.0526 (11)	0.0986 (17)	−0.0201 (10)	0.0120 (12)	−0.0203 (11)
N1	0.0552 (8)	0.0337 (6)	0.0348 (6)	−0.0014 (5)	0.0122 (5)	−0.0025 (5)
N2	0.0437 (7)	0.0397 (6)	0.0371 (6)	0.0010 (5)	0.0144 (5)	−0.0013 (5)
N3	0.0491 (8)	0.0403 (7)	0.0564 (8)	−0.0105 (6)	0.0097 (6)	−0.0086 (6)
O1	0.0412 (6)	0.0587 (7)	0.0583 (7)	−0.0084 (5)	0.0147 (5)	−0.0007 (5)
O2	0.0896 (10)	0.0578 (8)	0.0428 (6)	−0.0015 (7)	0.0112 (6)	−0.0112 (6)
O3	0.0682 (8)	0.0573 (7)	0.0379 (6)	−0.0059 (6)	0.0154 (5)	0.0073 (5)
Cl1	0.0575 (3)	0.0824 (4)	0.0885 (4)	0.0046 (2)	0.0407 (3)	−0.0143 (3)
F1	0.1338 (12)	0.0469 (6)	0.0633 (7)	0.0117 (7)	0.0138 (7)	0.0139 (5)

### Geometric parameters (Å, º)

**Table:**

C1—C2	1.370 (3)	C15—C16	1.448 (2)
C1—C6	1.391 (2)	C16—C17	1.342 (2)
C1—Cl1	1.7369 (18)	C16—H16	0.9300
C2—C3	1.385 (3)	C17—N1	1.3935 (18)
C2—H2	0.9300	C17—C18	1.478 (2)
C3—C4	1.379 (2)	C18—C19	1.385 (2)
C3—H3	0.9300	C18—C23	1.387 (2)
C4—C5	1.388 (2)	C19—C20	1.388 (3)
C4—N3	1.403 (2)	C19—H19	0.9300
C5—C6	1.374 (2)	C20—C21	1.367 (3)
C5—C7	1.508 (2)	C20—H20	0.9300
C6—H6	0.9300	C21—C22	1.366 (4)
C7—C24	1.521 (2)	C21—H21	0.9300
C7—C8	1.545 (2)	C22—C23	1.390 (3)
C7—C9	1.5489 (19)	C22—H22	0.9300
C8—O1	1.2107 (19)	C23—H23	0.9300
C8—N3	1.359 (2)	C24—N2	1.2926 (18)
C9—N1	1.4701 (18)	C24—C25	1.467 (2)
C9—C10	1.500 (2)	C25—O2	1.1981 (19)
C9—H9	0.9800	C25—O3	1.3308 (19)
C10—C11	1.385 (2)	C26—O3	1.440 (2)
C10—C15	1.405 (2)	C26—H26A	0.9600
C11—C12	1.375 (2)	C26—H26B	0.9600
C11—H11	0.9300	C26—H26C	0.9600
C12—F1	1.361 (2)	C27—N3	1.450 (2)
C12—C13	1.368 (3)	C27—H27A	0.9600
C13—C14	1.375 (2)	C27—H27B	0.9600
C13—H13	0.9300	C27—H27C	0.9600
C14—C15	1.400 (2)	N1—N2	1.3401 (17)
C14—H14	0.9300		
			
C2—C1—C6	121.66 (17)	C17—C16—C15	122.56 (13)
C2—C1—Cl1	119.58 (13)	C17—C16—H16	118.7
C6—C1—Cl1	118.76 (13)	C15—C16—H16	118.7
C1—C2—C3	120.61 (16)	C16—C17—N1	116.91 (13)
C1—C2—H2	119.7	C16—C17—C18	124.46 (13)
C3—C2—H2	119.7	N1—C17—C18	118.58 (13)
C4—C3—C2	118.03 (16)	C19—C18—C23	119.20 (16)
C4—C3—H3	121.0	C19—C18—C17	121.21 (15)
C2—C3—H3	121.0	C23—C18—C17	119.53 (16)
C3—C4—C5	121.20 (16)	C18—C19—C20	119.83 (19)
C3—C4—N3	129.00 (15)	C18—C19—H19	120.1
C5—C4—N3	109.79 (14)	C20—C19—H19	120.1
C6—C5—C4	120.81 (14)	C21—C20—C19	120.8 (2)
C6—C5—C7	130.34 (13)	C21—C20—H20	119.6
C4—C5—C7	108.66 (13)	C19—C20—H20	119.6
C5—C6—C1	117.69 (14)	C22—C21—C20	119.7 (2)
C5—C6—H6	121.2	C22—C21—H21	120.1
C1—C6—H6	121.2	C20—C21—H21	120.1
C5—C7—C24	111.26 (11)	C21—C22—C23	120.6 (2)
C5—C7—C8	102.06 (12)	C21—C22—H22	119.7
C24—C7—C8	114.32 (12)	C23—C22—H22	119.7
C5—C7—C9	120.38 (12)	C18—C23—C22	119.9 (2)
C24—C7—C9	98.91 (11)	C18—C23—H23	120.1
C8—C7—C9	110.56 (12)	C22—C23—H23	120.1
O1—C8—N3	126.20 (15)	N2—C24—C25	123.66 (13)
O1—C8—C7	125.94 (14)	N2—C24—C7	114.07 (12)
N3—C8—C7	107.83 (13)	C25—C24—C7	121.86 (12)
N1—C9—C10	111.48 (12)	O2—C25—O3	125.12 (15)
N1—C9—C7	101.95 (11)	O2—C25—C24	122.13 (15)
C10—C9—C7	120.01 (12)	O3—C25—C24	112.75 (13)
N1—C9—H9	107.6	O3—C26—H26A	109.5
C10—C9—H9	107.6	O3—C26—H26B	109.5
C7—C9—H9	107.6	H26A—C26—H26B	109.5
C11—C10—C15	120.27 (14)	O3—C26—H26C	109.5
C11—C10—C9	121.18 (13)	H26A—C26—H26C	109.5
C15—C10—C9	118.20 (13)	H26B—C26—H26C	109.5
C12—C11—C10	118.52 (15)	N3—C27—H27A	109.5
C12—C11—H11	120.7	N3—C27—H27B	109.5
C10—C11—H11	120.7	H27A—C27—H27B	109.5
F1—C12—C13	118.49 (16)	N3—C27—H27C	109.5
F1—C12—C11	118.17 (16)	H27A—C27—H27C	109.5
C13—C12—C11	123.32 (16)	H27B—C27—H27C	109.5
C12—C13—C14	117.94 (15)	N2—N1—C17	124.28 (12)
C12—C13—H13	121.0	N2—N1—C9	112.83 (11)
C14—C13—H13	121.0	C17—N1—C9	122.89 (12)
C13—C14—C15	121.58 (15)	C24—N2—N1	108.56 (12)
C13—C14—H14	119.2	C8—N3—C4	111.52 (13)
C15—C14—H14	119.2	C8—N3—C27	123.02 (16)
C14—C15—C10	118.36 (14)	C4—N3—C27	125.18 (16)
C14—C15—C16	121.55 (14)	C25—O3—C26	115.63 (14)
C10—C15—C16	120.02 (13)		
			
C6—C1—C2—C3	0.7 (3)	C14—C15—C16—C17	−172.17 (14)
Cl1—C1—C2—C3	179.82 (16)	C10—C15—C16—C17	10.8 (2)
C1—C2—C3—C4	0.1 (3)	C15—C16—C17—N1	−1.3 (2)
C2—C3—C4—C5	−0.5 (3)	C15—C16—C17—C18	−178.81 (14)
C2—C3—C4—N3	−179.09 (18)	C16—C17—C18—C19	134.10 (17)
C3—C4—C5—C6	0.1 (2)	N1—C17—C18—C19	−43.4 (2)
N3—C4—C5—C6	178.94 (14)	C16—C17—C18—C23	−43.1 (2)
C3—C4—C5—C7	−175.39 (16)	N1—C17—C18—C23	139.34 (16)
N3—C4—C5—C7	3.42 (17)	C23—C18—C19—C20	−0.1 (3)
C4—C5—C6—C1	0.7 (2)	C17—C18—C19—C20	−177.38 (16)
C7—C5—C6—C1	175.11 (15)	C18—C19—C20—C21	−0.4 (3)
C2—C1—C6—C5	−1.1 (2)	C19—C20—C21—C22	0.7 (3)
Cl1—C1—C6—C5	179.79 (12)	C20—C21—C22—C23	−0.5 (4)
C6—C5—C7—C24	−54.3 (2)	C19—C18—C23—C22	0.3 (3)
C4—C5—C7—C24	120.63 (13)	C17—C18—C23—C22	177.63 (17)
C6—C5—C7—C8	−176.67 (15)	C21—C22—C23—C18	0.0 (3)
C4—C5—C7—C8	−1.71 (15)	C5—C7—C24—N2	112.23 (14)
C6—C5—C7—C9	60.6 (2)	C8—C7—C24—N2	−132.82 (14)
C4—C5—C7—C9	−124.49 (14)	C9—C7—C24—N2	−15.38 (15)
C5—C7—C8—O1	−178.81 (15)	C5—C7—C24—C25	−60.72 (17)
C24—C7—C8—O1	60.97 (19)	C8—C7—C24—C25	54.23 (18)
C9—C7—C8—O1	−49.6 (2)	C9—C7—C24—C25	171.67 (13)
C5—C7—C8—N3	−0.59 (15)	N2—C24—C25—O2	179.97 (16)
C24—C7—C8—N3	−120.82 (14)	C7—C24—C25—O2	−7.8 (2)
C9—C7—C8—N3	128.63 (13)	N2—C24—C25—O3	0.6 (2)
C5—C7—C9—N1	−103.57 (14)	C7—C24—C25—O3	172.85 (13)
C24—C7—C9—N1	17.58 (13)	C16—C17—N1—N2	157.90 (14)
C8—C7—C9—N1	137.85 (12)	C18—C17—N1—N2	−24.4 (2)
C5—C7—C9—C10	20.10 (19)	C16—C17—N1—C9	−22.7 (2)
C24—C7—C9—C10	141.26 (13)	C18—C17—N1—C9	155.05 (14)
C8—C7—C9—C10	−98.48 (15)	C10—C9—N1—N2	−146.49 (12)
N1—C9—C10—C11	164.24 (13)	C7—C9—N1—N2	−17.24 (16)
C7—C9—C10—C11	45.3 (2)	C10—C9—N1—C17	34.01 (19)
N1—C9—C10—C15	−22.57 (18)	C7—C9—N1—C17	163.25 (13)
C7—C9—C10—C15	−141.53 (13)	C25—C24—N2—N1	178.35 (13)
C15—C10—C11—C12	0.4 (2)	C7—C24—N2—N1	5.55 (17)
C9—C10—C11—C12	173.48 (15)	C17—N1—N2—C24	−172.44 (14)
C10—C11—C12—F1	−177.63 (15)	C9—N1—N2—C24	8.07 (17)
C10—C11—C12—C13	0.6 (3)	O1—C8—N3—C4	−179.07 (15)
F1—C12—C13—C14	177.34 (16)	C7—C8—N3—C4	2.72 (18)
C11—C12—C13—C14	−0.9 (3)	O1—C8—N3—C27	−4.9 (3)
C12—C13—C14—C15	0.1 (2)	C7—C8—N3—C27	176.88 (16)
C13—C14—C15—C10	0.9 (2)	C3—C4—N3—C8	174.74 (18)
C13—C14—C15—C16	−176.19 (15)	C5—C4—N3—C8	−3.95 (19)
C11—C10—C15—C14	−1.1 (2)	C3—C4—N3—C27	0.7 (3)
C9—C10—C15—C14	−174.39 (13)	C5—C4—N3—C27	−177.97 (17)
C11—C10—C15—C16	175.96 (14)	O2—C25—O3—C26	−2.6 (3)
C9—C10—C15—C16	2.7 (2)	C24—C25—O3—C26	176.75 (16)

### Hydrogen-bond geometry (Å, º)

**Table:**

*D*—H···*A*	*D*—H	H···*A*	*D*···*A*	*D*—H···*A*
C27—H27*B*···F1^i^	0.96	2.52	3.226 (3)	130
C14—H14···O1^ii^	0.93	2.50	3.402 (2)	163

Symmetry codes: (i) −*x*, −*y*+1, −*z*; (ii) *x*−1/2, −*y*+1/2, *z*−1/2.
